# **Aiming for Health Equity: The role of Public Health Policy and Primary Healthcare** Comment on "Universal Health Coverage for Non-Communicable Diseases and Health Equity: Lessons From Australian Primary Healthcare"

**DOI:** 10.34172/ijhpm.2021.112

**Published:** 2021-09-01

**Authors:** Bo Burström

**Affiliations:** Department of Global Public Health, Karolinska Institutet, Stockholm, Sweden.

**Keywords:** Inequalities in Health, Equity in Healthcare, Health Policy, Primary Healthcare

## Abstract

This commentary refers to the article by Fisher et al on lessons from Australian primary healthcare (PHC), which highlights the role of PHC to reduce non-communicable diseases (NCDs) and promote health equity. This commentary discusses important elements and features when aiming for health equity, including going beyond the healthcare system and focusing on the social determinants of health in public health policies, in PHC and in the healthcare system as a whole, to reduce NCDs. A wider biopsychosocial view on health is needed, recognizing the importance of social determinants of health, and inequalities in health. Public funding and universal access to care are important prerequisites, but regulation is needed to ensure equitable access in practice. An example of a PHC reform in Sweden indicates that introducing market solutions in a publicly funded PHC system may not benefit those with greater needs and may reduce the impact of PHC on population health.

 Fisher et al^1^ report on a policy analysis of primary healthcare (PHC) implementation in Australia from the perspective of universal health coverage (UHC), its strengths and weaknesses in addressing non-communicable diseases (NCDs), and how it contributes to equity in access to care and in health outcomes. They examine four key issues: (*a*) the role of public or private funding or services delivery; (*b*) PHC responses to the growing impacts of NCDs; (*c*) utilization of comprehensive, selective or medical models of primary care; and (*d*) the extent to which UHC systems in practice will support universal and equitable access to PHC and equity in health outcomes.

 Drawing on their results, the authors propose some recommended generic UHC features for PHC systems regarding ideas, actors and structures. They distinguish between PHC and primary medical care, and propose PHC, which has a wider scope, encompassing also health promotion, disease prevention, community engagement and actions to address social determinants of health.^2^ Fisher et al^1^ find that the Australian system is currently based on a narrow biomedical and behavioural view of health and propose a wider approach based on a broader biopsychosocial model of health, recognizing the influence of other social and commercial determinants of health. They also recommend commitments to principles of universal access according to need, health equity and prevention. Fisher et al propose policy decision-making processes that limit the influence of certain groups with financial interests, eg, medical professionals, private health insurance and the tobacco, food and alcohol industry. When it comes to structures, the recommendations include a public scheme, funded by progressive taxation, committed to equity of access to all services; regulatory measures and incentives to ensure that services match the needs; block funding of multidisciplinary services which can be adapted to local conditions, with coordinated care, engaging local communities, also catering for minority groups. They also propose a multi-disciplinary PHC workforce, with secure employment conditions, supplementary funding according to need in certain areas. Other features should be public health regulation to limit the impact of the food, alcohol and tobacco industry on NCDs, and regional PHC organisations for health planning and workforce planning, to ensure coordination between different levels of services and address social determinants of health.

 Risk factors of NCDs (eg, smoking, alcohol and obesity) require regulation also of commercial actors,^3^ which may be a sensitive political issue. However, reducing the impact of NCDs, cannot be solved only within PHC or healthcare service as a whole – it requires the involvement of other societal sectors, and policy making with a road map and a joint plan for the different actors on how to collaborate.^3^ This is true not only for NCDs, but also for many other health problems. One important basis for a country’s health policy and healthcare system is the prevailing view of health and the importance of social determinants of health. With a predominantly biomedical view on health as described in the Australian study, and seeing health only as a personal responsibility, the role of other important social determinants will be underestimated and the potential to improve health will be reduced. Similarly, health inequalities and health in disadvantaged groups may not be recognized and focused for action. In order to address NCDs and reduce inequalities in health it is important to combine policy efforts to reduce risk factors on a population level with efforts in healthcare services to prevent, detect and manage manifestations of NCDs. PHC is the first point of contact with healthcare services for most individuals and has a fundamental role in this respect. PHC services should be designed to handle both prevention and management of disease.

 The study by Fisher et al^1^ is an interesting account of the Australian healthcare system, but also reflects more general features and drivers of healthcare systems – what is the ideal, and what is the reality? Many countries aim for universal access to healthcare in their health policy documents, that services should be provided equitably and according to need. However, in reality this is seldom completely achieved. The reasons for this may vary, but as indicated by Fisher et al, some features may be generic and need to be addressed in many countries.

 Fisher et al outline some important building blocks for an equitable healthcare system. A strong, well-resourced, publicly funded PHC system, accessible for all, has been recognized by many,^4^ including the Organisation for Economic Co-operation and Development^5^ to be a good foundation for PHC, which can handle a large part of common health issues, and contribute to reducing health inequalities.^6^ Funding and access to care are important to achieve an equitable healthcare system. Fisher et al^1^ propose a public scheme, funded by taxation, committed to equity and with regulation and incentives to ensure a distribution of resources and staff according to need. This is vital for PHC and for the health system as a whole. Market forces can seldom improve problems of equity in access to care. The well-known quote by Julian Tudor Hart regarding the ‘Inverse care law’ from 1971^7^ still holds true: “The availability of healthcare tends to vary inversely with the need for it in the population. This inverse balance operates more completely where healthcare is most exposed to market forces, and less so with less exposure.”^7^ Access to care in remote areas is an illustrative example. As reported in the Australian study,^1^ and as observed in many other settings, the fact that there is no regulation in the system to control the distribution of PHC services leads to concentration of services in urban areas and an undersupply in rural areas. Ensuring equity in access to PHC also in sparsely populated areas seems to require regulation of establishment of services, and incentives making it attractive also to work there. As demonstrated in the Australian study,^1^ private health insurance does not contribute to equity in health or healthcare.

 In many high-income countries, older persons with multiple chronic diseases sometimes also with complex health and social care needs, are major users of healthcare.^5^For this group, it is particularly important that the care provider has a holistic view of health and of the patient, that PHC can provide multidisciplinary services and be a link to other actors, for instance to social services, as also suggested by Fisher et al.^1^

 NCDs are a major health issue also in many European countries. Countries address common health problems (eg, obesity and alcohol) in different ways, some to a large extent using policies addressing actors outside the health sector, including the food and alcohol industry.^8^ Alcohol and tobacco policies, including increasing prices via taxes, restricting availability and marketing may be effective, and are used in many European countries.^8^ Such policies also contribute to reducing inequalities in health. Sweden has a National Public Health policy focusing on the social determinants of health and reducing inequalities in health, recognizing the importance of other sectors of society, and of the impact on health of regulation and other policy measures outside the health sector.^9^ The National Public Health policy further advocates for an equitable and more health-promoting healthcare system, with an emphasis on prevention and health promotion efforts, recognizing and targeting groups with greater needs.^9^ However, putting the policy on equitable and health-promoting healthcare into practice is not easily done.

 The Swedish healthcare system is universal, tax-funded and largely publicly provided, equity in health and healthcare is high on the agenda.^10^ The responsibility for the provision of healthcare lies with the 21 regions, which also collect taxes. The national level is responsible for legislation and supervision, and provides some special grants, PHC is a basis for the healthcare system, and should be the first point of care for the population.^10^ However, only about 15 per cent of all doctors work in PHC, and as noted in the Australian study, other types of health services tend to get a larger share of resources. Nevertheless, most persons with chronic diseases including NCDs are managed in PHC.

 In order to increase access to PHC, a reform was enacted in Sweden 2010, allowing free establishment of for-profit operating PHC clinics with public funding, and free choice of providers among the public, with a voucher system.^11^ The reform limited the ability of the public purchasers to distribute resources to PHC clinics based on need or location.^12^ The new providers therefore established themselves mainly in already well-served and affluent urban areas, less in disadvantaged and rural areas.^11,13^ The reform further shifted the focus of PHC from having an area level responsibility for the health of the population served, to focusing only on those individuals listed with the particular clinic, thereby reducing the potential impact of PHC on population health. The funding mechanisms and design of reimbursement systems in PHC were also changed.^12^ A recent qualitative interview study among PHC doctors in Sweden showed that the doctors are very much aware of the reimbursement systems, and that a fee-for-service system incentivised shorter visits of healthier patients, to increase revenues.^14^ There has also been accounts of less emphasis on health promotion and prevention in PHC.^12^ The fee-for-service based reimbursement system in PHC in the Australian study also led to separate episodic care,^1^ which is not optimal for managing and preventing NCDs. Changes in reimbursement systems in healthcare may have unintended consequences, and it is important to consider their advantages and disadvantages.^15^ The experience of the Swedish reform shows that market solutions in PHC may have definitive downsides.

 When aiming for health equity it is important to go beyond the healthcare system, including focusing on the social determinants of health in public health policies and in the healthcare system. PHC is an important part of any country’s healthcare system and can have a central function as being close to the patients and the population served, being a link to the other parts of the healthcare system as well as to other sectors in society. PHC has the potential to improve the efficiency of healthcare systems and to reduce health inequalities, recognizing and addressing the social determinants of health. However, as indicated in the Australian study,^1^ it is important that the funding of PHC ensures universal and equitable access to PHC and that there is regulation to achieve this also in less populated areas and among disadvantaged groups. Learning from international comparisons and the experiences of other countries may be useful.

## Ethical issues

 Not applicable.

## Competing interests

 Author declares that he has no competing interests.

## Author’s contribution

 BB is the single author of the paper.
